# Condoms, Trust and Stealthing: The Meanings Attributed to Unprotected Hetero-Sex

**DOI:** 10.3390/ijerph18084257

**Published:** 2021-04-16

**Authors:** Najiha Alam, Pam Alldred

**Affiliations:** 1 Department of Clinical Sciences, Brunel University London, London UB8 3PH, UK; naalam@ucdavis.edu; 2 School of Social Sciences, Nottingham Trent University, Nottingham NG1 5LT, UK

**Keywords:** sexual health, condom use, gender inequality, stealthing, interpersonal violence (IPV)

## Abstract

University students tend to have greater sexual health knowledge than the general public, yet condom use among this group continues to be a public health concern because effective condom use could reduce sexually transmitted infections and, for heterosexual women, unwanted pregnancies. We report findings from a small, qualitative study of condom use among sexually active heterosexual university students in the UK. In interviews, students shared their views about condom use and sometimes their personal experiences too. This paper identifies some of the meanings attributed to condom use in the accounts of nine heterosexually active 20–25 year-olds. Participants explained that when they felt comfortable communicating with their partners, they were more likely to use condoms, and those with negative sexual experiences or under social or psychological pressure were less likely to use them. The findings highlight issues of trust and power between men and women in heterosexual relationships, and describe contexts for dishonest sexual practice, including the traditional notions of femininity that were linked to condom use by this group. The issue of stealthing arose in one woman’s account of her experience and in several others’ reports of what occurs commonly. Stealthing, the secretive removal of a condom by a (usually male) partner during sexual intercourse without a partner’s knowledge or permission, produces non-consensual unprotected sex. We present stealthing as a product of the sexual double-standards described and as a form of interpersonal violence (IPV) and, among these heterosexual partners, as a form of gender-based violence. This study provides a glimpse into university students’ decision-making regarding condom use and highlights how gendered inequalities shape heterosex, in particular, communication about safer sex, that in some cases, compromise women’s decisions about (safer) sex.

## 1. Introduction

Inadequate condom use is linked to higher rates of terminations and sexually transmitted infections (STIs) and when acquired in youth, STIs can jeopardise sexual and reproductive health later in life and, for women, the health of babies [1]. Young people’s sexual health is therefore an important public health concern, with generational implications. One demographic of young people, university students, are more likely to have knowledge about sexual health risks than the general public [2,3,4,5,6]. However, university students continue to have relatively high rates of unplanned pregnancy and STIs [7]. Having higher levels of education and of unplanned pregnancy/STIs highlights that knowledge is necessary, but not sufficient, for safer sexual practice and that more research is needed to understand this group’s behaviour.

This study set out to identify the barriers to and facilitators of condom use amongst young sexually active heterosexual university students in the UK, but in documenting talk about condom use, we now frame this as a study of the meanings attributed to using or not using condoms. The research was conducted at an outer London university, for a Masters in Health Studies and was granted ethical approval (on 12 August 2019) by the university at which it was conducted (Brunel University London, UK).

## 2. Background

In the literature on intentions and barriers to condom use, SCOPUS and Web of Science identified a systematic review and 13 relevant quantitative studies. Of the 13 studies, nine were conducted in Western countries (the United Kingdom, United States of America and Canada) and four in non-Western countries (two in Africa and two in China) between 2012 and 2016. In these 14 articles, four types of factor are found to impact condom use: psychosocial; sociocognitive; environmental; and economic and structural factors. Environmental factors, like access to condoms, clearly restrict access, as do economic and structural issues like poverty and the legality of young people buying condoms. Sociocognitive issues include gender roles, peer and parental norms, societal stigma, whether it is a steady or casual partner, and whether the context is a collectivist or individualistic culture. Psychosocial factors include confidence and self-efficacy, shame, embarrassment and guilt, loss of pleasure, comfort communicating with partners, lack of emotional intimacy, and an association with impurity or lack of trust.

Although health behaviours are enacted at the individual level, they are shaped on multiple macro levels and the choices available to the individual are also shaped by the structure and norms of that society. Thus, sexual health decisions are shaped by a complex interaction between psychosocial, sociocognitive, environmental, and structural factors, which vary between different contexts and societies [8,9,10]. Although common factors can be seen across location, impact on condom-use decision-making may vary between more collectivist or individualist (Western) societies [11]. For the studies of non-Western contexts, perceived social norms (i.e., sociocognitive factors like perceived peer norms, stigma, taboos, etc.) were a stronger predictor of condom use than self-efficacy and positive attitudes toward condoms. In contrast, studies in Western contexts found psychosocial factors (e.g., self-efficacy and positive attitudes) were the strongest predictors of condom use.

Another distinct difference in condom use concerns behaviour with casual partners versus with long-term partners. In Western contexts, studies found that condoms were used most frequently with casual partners [12,13,14] whereas studies of non-Western contexts found condom use more common with steady partners [15,16]. This difference may be explained by different norms of partner communication. For example, some argue that the “conservative” nature of Chinese relationships may hinder individuals’ ability to discuss condom use openly, thus it is avoided as affectively costly, although this cost is more likely to be borne in relation to steady partners [16]. In contrast, studies in the West find that condom use is avoided with long-term partners, as it is thought to imply reduced emotional intimacy or trust [12,13,14,17].

Common trends are also apparent across contexts: women tend to have more favourable attitudes toward condom use than men [17,18] (only binary gender categories were considered in these studies unfortunately). However, this did not mean that women were more likely to use condoms. In fact, Wendt and Solomon [19] found that women were highly likely to be-non-users, a trend later reaffirmed by Nesoff et al. [20]. Gender roles impact how able women feel to discuss condom use or to buy and carry condoms [11], and there are degrees of societal barriers to women taking control of their own sexuality, for instance, by using condoms, in both Western and non-Western contexts. Therefore, women’s favourable attitude toward using condoms may not necessarily mean that they use them [21].

Greater knowledge of the risks of unprotected sex is associated with a greater likelihood of condom use, but previous studies suggest that health knowledge is necessary but not sufficient to lead to behavioural change [18,22]. This may be explained by the distinction between cognitive responses—the thoughts or beliefs surrounding a subject—and affective responses—the emotions evoked by the subject. Positive cognitive responses to condom use are likely in a wide range of cultural contexts, since condom use is associated with reducing health risks [13,14,15]. However, affective responses to condom use may vary more widely and can prompt aversion to them [14]. Aversion to condoms may be explained by a variety of factors, including discriminatory views among heterosexuals like association with homosexuality, stigma related to HIV, and perceived impurity, to a perceived reduction of pleasure [14,17,23]. As these affective pressures exist in both Western and non-Western contexts, the same trend of inconsistent condom use irrespective of sexual health risk knowledge emerges. Additionally, the one-time stigma related to condom use has shifted. In pre-2000 studies in the West, shame, guilt and embarrassment associated with condom use was partially attributed to the stigma related to homosexuality [18] whereas more recent studies suggest shame, guilt and embarrassment associated with condom use centre on the perception that their use implies a lack of trust in a partner [22,24]. We are particularly interested in this and other psychosocial factors.

Common among all the studies as significant predictors of non-use of condoms were the perception that condoms reduce sexual pleasure, and the issue of communication: the more comfortable young people feel discussing condom use, either with family or partners, the more likely they are to use them [11,19]. Comfort communicating about condom use is impacted on by social norms, and in patriarchal societies gender roles and expectations include sexual double standards in heterosexual relationships.

“Stealthing”, the practice of non-consensually removing condoms during sex, has become a more common, serious and widespread issue in recent years [25]. Research on stealthing has been published in the last five years, but the earlier literature is very sparse, suggesting that it is either a relatively recent issue or only more recently identified. Studies have suggested that stealthing is associated with greater hostility, more severe sexual aggression history, and characterized by disrespect and selfish behaviour of men toward women [26,27]. Men with a history of stealthing behaviour are significantly more likely to have had an STI or have had a partner who experienced unplanned pregnancy [26]. Although not formally recognised as rape, it corrupts the conditions in which sex was consented to and is recognized as a form of interpersonal violence (IPV).

This (cross-sectional survey) literature evidences associations between aspects of sexual behaviour, which of course is not causality. Self-reported sexual behaviour (over the previous 6–12 months) could have reflected perceived social desirability and be subject to recall bias. This study seeks to reduce the impact of social desirability by inviting discussion in the third person by using vignettes, and although we cannot eliminate participants’ potential self-consciousness of how they present themselves, this may have reduced the degree to which their responses were shaped by this.

## 3. Method and Approach

Semi-structured interviews using vignettes were used to explore perspectives on and experiences of condom use of sexually active heterosexual university students in the UK. The aim was to identify the barriers to and facilitators of condom use among this group. University students at one outer London university were invited to participate on flyers that were distributed across campus, as well as online, including via Facebook, in the hope of incorporating diverse participants. Students interested in opting into the study emailed the first author, herself a Masters student at the university, and were sent the Participant Information Sheet to read before deciding whether to participate.

Nine university students took part in the study—enough to offer insight into some perspectives and demonstrate some of the dynamics of condom use—exploring “how” rather than “how much” [28,29]. Inclusion criteria were: being a university student, young—by the British Youth Council [30] definition of up to 25 years—and having had heterosexual intercourse within the last two years. This time frame (i.e., within the last two years) aimed to reduce recall bias [31].

Interviews were conducted and audio recorded in the university library. As sexual practice is a sensitive topic, the interviews began with the discussion of vignettes, which offered short scenarios about hypothetical characters to glean information about participants’ beliefs without asking directly about their own experience [32,33]. The vignettes described various scenarios, in either the context of a hook-up or an ongoing relationship, where a heterosexual couple engaged in intercourse without a condom. Participants were asked to describe what the thought process of the character might have been in each scenario and what could have led to the outcome of engaging in unprotected sex. Different vignettes were offered according to participants’ declared gender and then, depending on responses, questions were followed by prompts, if needed. As the focus is on a third person, vignettes can help participants feel comfortable when they might not feel comfortable discussing themselves and may conceal their own actions or beliefs [33]. After discussion of the vignettes and once the researcher was confident that rapport had been established, participants were asked if they would comment on what they thought they would do themselves, so the conversational flow directed the interview and dictated the focus. Through the discussion and normalisation of the scenario, vignettes encourage participants to share their views or their own personal experiences if and when they feel comfortable to do so.

Although sensitive topics were discussed, at no time before, during or after the interview, did any of the participants indicate that they felt uncomfortable or wanted to discontinue. At the start of the interview, they were reminded that they could pause or halt the interview, as had been stated on the Participant Information Sheet. It was hoped that the conversational nature of this method would allow the interviewee a more confident role and facilitate a more ethical and accurate process of knowledge production [34].

All the interviews were manually transcribed. Transcripts were then analysed thematically by the first author using Braun and Clarke’s [35,36] six step framework of data emersion, initial coding of individual items, identification of themes among them, then review and mapping of all codes against the themes. A reflexive approach means acknowledging that the themes identified reflect the feminist principles that shaped interest in the topic originally, such as a concern with how gender dynamics affect the negotiation of sexual health (e.g., [37]).

## 4. Findings

Of the nine participants, three were male and six female, with an average age of 22.6 years. Six were post-graduate and three were undergraduate students. Interviews ranged from 15 to 30 min and were all conducted by NA1. A thematic analysis led to the identification of six themes, each with multiple subthemes, which Table 1 summarises.

### 4.1. Experiences and Education

Among all participants, condom use behaviour was influenced by the amount of sex education and sexual experience they had had. Almost all reported that formal sex education was not comprehensive enough to facilitate condom use and, instead, their personal experiences or their friends’ experiences were key factors in their competence and knowledge regarding condom use. They agreed that pregnancy prevention was emphasized in lessons, leading them to underestimate the importance of condoms in preventing STIs. In some cases, this acted as a barrier to condom use. One female student, Participant 4, said: “*I honestly feel like, growing up with the sex ed that I had, if they put an emphasis on the STIs you can get, I would have been definitely more scared…if they had taught us like, ‘Yeah, you can [get pregnant], but you can also get Gonorrhoea or Herpes’, I would have been like, ‘Oh, shit. Really?’. Yeah, it was only emphasised how you can get unwanted pregnancies*”.

As a consequence, participants did not have condoms accessible for when the need arose and would therefore have intercourse without protection: “*I’m not gonna lie. Each time, it was the same… like the scenario that you showed me where it is spur of the moment. There was no time for us to like get a condom, so I just did it*” (Participant 4).

Instead of formal education informing their decisions, participants agreed that their own prior experiences, whether through previous personal experience or second-hand experience, and particularly witnessing the risks of not using condoms, was key to their sexual health knowledge and future condom use. As Participant 4 (a female student) shared: “*I had a pregnancy scare and it really made me think about things. [Since] that situation, I insist ‘Oh, can you please put on a condom?’*” and as Participant 2, another female student, said: “*I got checked up …after him and yeah, he had given me Chlamydia…So after that, I was just kind of like, ‘No, dude, I cannot do it without one’*”.

Many participants recognised the importance of taking the initiative to educate themselves about sexual health, so that they could make informed decisions. For instance:

“*[My] research started off with what type of birth control I wanted, and there’s a really good website called bedsider.org that I always refer to [which has a] comprehensive understanding of all the birth control available. It was there I realised that there’s risk for all these different STIs… but what is out there? That’s when I used to Google different STIs…How do you get Chlamydia? How do you get Herpes? What’s treatable? What’s not treatable? I just kind of went on to like my own rabbit hole with that*”

(Participant 4, female student)

Amongst the women participants there was a strong view of a correlation between their sexual experience and the likelihood of them voicing their desire to use condoms. They described how, when they were younger and less experienced, they felt less comfortable voicing their concerns about unprotected sex to partners. With more sexual experience they were more likely to voice discomfort and their condom preferences. Therefore, age and sexual experience were identified as facilitators for condom use. The following comments illustrate what we view as the internal silencing of women: “*the first time, I didn’t use a condom… Your perspective changes as you grow older. I feel like once you make those mistakes, you don’t really do it again when you’re getting older*” (Participant 5, female student) and “*After more sexual partners, I feel more comfortable saying how I want my body to be treated. As opposed to [being] very inexperienced and not knowing what to do, not knowing what to say, not knowing what’s appropriate, and not knowing how the other person would react*” (Participant 8, female student). Participant 4 (female student) also commented:

“*I feel like it really comes with experience. Because I was like that when I was younger… There was kind of intimidation to not ruin the mood by not saying what I wanted … whether I liked or didn’t like something.… I always felt scared that if I changed my mind, I would ruin the mood. Obviously, with experience… learning how to speak up and not be afraid. I’m more confident [But] it took a long time for me to realize [that], even if I agree to something, I shouldn’t just go through it because I didn’t want to disappoint the other person”.*

Participants described their sexual health knowledge being profoundly shaped by friends’ experiences. They were more likely to use condoms if they had heard about friends having negative experiences as a result of not using them. Thus, negative friends’ experiences were identified as a facilitator of condom use, as Participant 8 commented: “*I think my friends’ experiences definitely plays the biggest part [in informing my sexual health knowledge]*”, and Participant 7 (male student): “*some things you’re taught in school, but others you learn over time by hearing about them from friends*”.

### 4.2. Perceptions of Condom Purpose

Although all participants were aware and understood the dual purpose of condoms as contraceptive and prophylactic, they associated condoms with one purpose more than the other. Depending on which purpose participants’ prioritized affected how likely they were to use them. Regardless of which purpose they associated condom use with, participants would not use condoms, if they perceived their partner to be uninfected. When participants perceived condoms as primarily for contraception, as half of them did, they were less likely to use them, as they began to employ other forms of birth control and perceived their partners to be uninfected. For instance, Participant 4 (a female student) said: “*I was on top of the condom stuff, but now that I’m on birth control pills, I don’t have to worry about it. But I still have to remind myself that it’s not just pregnancy you have to worry about*”, and Participant 3 (a male student): “*I usually don’t really think about a condom from the STI side, because usually the people that I am sleeping with like I know what’s going on with them. So for us, [using condoms] would be more like a contraceptive type thing*”.

The half of the participants who associated condom use more with STI prophylaxis, were more likely to use them. However, exceptions were made and condoms not used if a participant trusted their partner/s and believed them to be uninfected. The complexity of the decision and judgements reflecting trust and perceived risk are illustrated in Participant 2 (a female student)’s comment:

“*For me it’s more of an STI prevention thing. Like definitely it’s [contraception] too, but I feel like if I want contraception, I would get birth control pills. Yeah, I don’t like depending on someone else [for STI prevention]. At least with a condom, I can see it, and I [know] I’m not going to get an STI. Because [with] birth control pills, you can still get [STIs]”.*

### 4.3. Communication

All participants stated that communicating with their sexual partners about their sexual health and desires was a major factor in determining whether they engaged in unprotected sex. When communication was good, they were more likely to make informed decisions about condom use. When they judged that this conversation would be distressing, they chose not to voice their preference, usually resulting in unprotected sex. Therefore, discomfort discussing condom use with sexual partners is a barrier to condom use.

#### 4.3.1. Trust and Comfortability with Partner

All participants agreed that trust was a major factor in facilitating communication with their partners. In all cases when participants reported trusting their partners, they discussed their sexual health concerns and made informed decisions on condoms use. Trust was more influential in facilitating condom use amongst casual relationships, as participants felt more comfortable discussing their sexual practice with their partner, concluding that condom use was preferred when either party had more than one sexual partner. In contrast, when reporting “trusting” their partners, they were less likely to use condoms, because they “trusted” that their partner did not have an STI and was monogamous. This judgement was made on the basis of “character” and length of their relationship. For instance, as female student, Participant 9, put it: “*She probably just trusts her partner… I’d say it’s probably she’s been with this person for a while and she knows them, [so] she probably just thinks that [unprotected sex] is right*.” Participant 7 (a male student) said: “*For me to [have unprotected sex], we talked about it, and I felt like I could trust her, right?*” and Participant 6 (a male student): “*when you’re in a relationship, you tend to trust what your partner is saying. So if they [vignette characters] decided to have sex without a condom…they came to an understanding that neither of them have STDs, so decided there was no risk*”.

#### 4.3.2. Lies and Misguided Trust

An important finding in this subtheme is that trust can be violated. Amongst the male participants, it was stated that lying to partners about wearing a condom is common in unprotected sex. Although the participants themselves had never lied about wearing a condom, they had heard stories that lead them to believe that this occurred often. Amongst the female participants, two raised lying and misguided trust as factors for non-condom use, and one indicated that this had happened to her. This illustrated the gendered power dynamics between male and female sexual partners at their worse. This had occurred to one of the female participants but was not reported by male participants to be a concern of theirs. Only women participants mentioned a concern that their trust in a partner could be misguided and they could have been lied to.

Participant 2 commented: “*They [vignette characters] either could have talked about it, or she [knew] him long enough to trust him. I feel like when you have one partner, even if you don’t know what they have, if you trust them enough, people fall into [unprotected sex]*” and Participant 9 (a female student) said: “*People lie in relationships all the time. People cheat relationships all the time or they could have picked up something in a previous sexual encounter and not know it*”.

Participant 6 (a male student) reported a troubling norm: “*[Lying doesn’t occur] among my group of friends. But, I’ve definitely heard of it a lot. So maybe… they were in a scenario where [he] lied about it, to not use [the condom]*” and Participant 7 (a male student) said: “*[vignette character] could have lied to her, and he didn’t use them…that could be one scenario. I’m not sure if it’s a common thing but I’ve heard about it a lot*”.

The most telling report was Participant 4’s account of her experience:

“*This one guy was my friend. We were on-and-off fuck buddies, and I always wore a condom with him. Last time, I literally handed him the condom and midway through, I noticed [it felt] a little different, and he was like ‘Don’t worry about it’. It turns out he took off the condom in the middle of sex, and I got so mad. I was like ‘What the fuck, I don’t know what the hell’s happening with you, but I want to stay protected… that’s the only reason why I asked you to wear a condom’. And, he was like, ‘Oh, but I like being defiant. I like not listening to you’. I’m like, that’s not the fucking point. When you take it off during sex without my knowledge, I feel violated. It was the weirdest situation I’ve ever been in, but it just made me like realize like some guys just don’t give a shit if I am trying to be protected*”.

The likelihood of communicating their sexual health preferences to their partner depended on how approachable they perceived their partner to be, having more conversations when they perceived their sexual partners as “friendly” and “approachable”. When participants felt their partners to be “unapproachable” or “aggressive”, they would not voice their concerns and would end up having unprotected sex. This appears to be a form of internal silencing where women decide it is “better” (perhaps safer) not to ask. For instance, Participant 8 (female student) said: “*For me, I think it is a matter of personalities. If I felt that they would be okay with me telling them to put on a condom, I would feel comfortable voicing that. But if they had a bolder or aggressive personality, I don’t think I would*” and Participant 1 (female student) said: “*Maybe it because [she] judged his character and decided to avoid any troubles that would come up if she brought up condom use*”.

In casual, hook-up relationships, participants immediately discussed their condom preference. Participants who engaged in casual sex wore condoms most. However, once casual partners established greater trust with their partner, they were more likely to engage in unprotected sex. By contrast, earlier on in *romantic* relationships, participants were less likely to discuss their preferences in order to indicate their investment in the relationship. Later on in romantic relationships, once trust, intimacy, and commitment were established, participants were more likely to communicate their sexual health concerns and desires. However, participants were less likely to use condoms in longer romantic relationships, as they trusted their partners more.

### 4.4. Social and Psychological Pressures

Social and psychological pressures were identified as key barriers to condom use amongst participants. Psychological pressures included perceived hurtful partner reactions, guilting, and fear of “ruining the moment”, which were all barriers to condom use. Social pressures included stigmas and stereotypes, which facilitated condom use in some cases but not others. What is meant by “Perceived Hurtful Partner Reactions” is the expectation that their sexual partners would react antagonistically to their request to use condoms, which all female participants mentioned, saying that they were reluctant or afraid to raise the subject. This perception was informed by social media and friends’ experiences. These illustrate an internal silencing that all the women participants shared. For instance, Participant 1 said that: “*By bring up like, ‘Oh, I’d prefer you put on a condom’, it would kind of put him off*”, Participant 8 described a “*fear of being rejected or made fun of [by] her male partner being a good reason as to why a lot of women do not say no to unprotected sex. Hearing what friends say or what the media portrays, I sense that it’s a common response*” and Participant 9 referred to it as: “*intimidating*” *asking for condoms, anticipating that they’re going to react negatively to it*”. In a few cases, when participants voiced their desire to use condoms, partners reacted by attempting to guilt trip them into engaging in unprotected sex. This psychological pressure made it more difficult for participants to remain resolute. Only female participants described being made to feel guilty by partners for wanting to use condoms, and this was more often with a casual partner.

Every participant referred to the pressure not to “ruin the moment”. Most participants felt that discussing condoms with their partners would be awkward, spoil the dynamic, and distract their partners from wanting intercourse. Due to this fear, participants would remain passive, “go with the flow”, and not discuss condoms. Fear of ruining the moment was a clear barrier to condom use. A few participants believed that the fear of ruining the moment was overstated, but all indicated that it was a significant pressure for them, and every male participant stated that they would still voice their concerns, while female participants were more likely to remain silent “*[because] you don’t want to ruin the mood*” as Participant 4 (female) said, and: “*Out of fear of bringing it up [and] like not wanting to ruin the moment. Just wanting [it to be] pleasurable and avoid[ing] any troubles that would come up if [she] had brought up condom use*” as Participant 1 (female) put it. The pressure of ruining the moment was not just limited to having conversations around condoms. In cases where participants and their partners had agreed beforehand to use a condom, the process of putting on the condom could also “ruin the moment”, leading participants to agree to unprotected sex with a partner in future.

Stereotypes around promiscuity imply that “the promiscuous” are more likely to have STIs. This created pressure for participants to use condoms, and all participants preferred them when they knew their partner might have more than one sexual partner. However, there was a gender difference: male participants were more likely than female participants to stereotype promiscuous partners based on superficial characteristics rather than actual behaviour: “*Wearing condoms depends on if I hundred percent trust that my partner is faithful*” (Participant 5, female) and “*If I’m hitting on a chick at a library, that’s a different scenario, if you’re hitting on a chick at the club … I mean, it’s an assumption that girls at the club sleep around a lot more*” (Participant 6, male).

### 4.5. Decision Making Effort

The amount of effort that participants put into condom-use decisions influences the likelihood of using them. When participants took time to consider many factors, including long-term consequences of non-use, they were more likely to use condoms. When participants did not think critically about safer sex, they were less likely to use them.

#### 4.5.1. Subtheme: Comprehensive Decision Making

Participants were more likely to use condoms when they took time to consider the consequences of non-condom use. Common factors considered were: the emotional investment of their partner in the relationship, the likelihood that their partner did not have an/other sexual partner/s, fear of a hurtful partner reaction, if discussing condom use would ruin the moment, and if they used another form of birth control. Many participants conducted a cost–benefit analysis to determine if the benefits of not wearing a condom with their partner would outweigh the costs. All participants considered that the benefits outweighed the costs when they were in long-term, intimate, romantic relationships. This was in contrast to hook-up or casual relationships, where most participants determined that the risk of STIs outweighed the potential benefits of unprotected sex.

“*[Before we had unprotected sex], we considered, in our relationship, our commitment and our trust in each other… our ability to trust that our partner hasn’t had sex outside of our relationship, because that would pose a lot of risk to me and him, as well. I think people tend to think that, in their situation, that risk might not be applicable or might be so minimal that it wouldn’t matter if ‘this once’ they didn’t use protection*”

(Participant 8, female)

#### 4.5.2. Subtheme: Lack of Critical Thinking

Participants are more likely to have unprotected sex when they do not take time to consider the consequences. In every case where participants were “in the throws of it”, they did not think critically about their behaviour, and had unprotected sex. Some were in long-term relationships, so had considered the risks previously and decided that unprotected sex was “not too risky” (e.g., see Participant 9 in 4.5.1. Comprehensive Decision Making). However, it was more common for participants to engage in unprotected sex without any consideration at all, as they were swept away by desire in the heat of the moment. In almost all cases, participants’ lack of critical thinking was attributed the impaired judgment caused by alcohol consumption: For example: “*Where it’s a heat of the moment thing some people just succumb to their emotions and their hormones and, once they start, they can’t stop [to consider anything]*” (Participant 7, male), and “*See the problem is there’s alcohol involved, so nobody’s really making really informed decisions*” (Participant 3, male).

### 4.6. Theme: Pleasure

The most common barrier to condom use is “pleasure”. Almost all participants, (so including women students), agreed that not using condoms provides greater pleasure, both physical and emotional. Physical pleasure was the most impactful in the decision to not wear condoms, but many participants preferred not using condoms for emotional pleasure too.

#### 4.6.1. Subtheme: Physical Pleasure

All participants agreed that not wearing condoms provided greater physical pleasure and this was the greatest facilitator of unprotected sex. This comment was typical: “*But I know for a fact like, just like physically, I feel raw sex is better than like protected sex, because it feels better*” (Participant 7, male). In a few cases, participants shared accounts of how the physical discomfort of putting on a condom, for them or their partner, prevented participants from engaging in sex, thus motivating their decisions not to wear condoms. However, occasionally, participants reported the discomfort of wearing a condom as a benefit, as they lasted longer during sex.

#### 4.6.2. Subtheme: Emotional Pleasure

Almost all participants agreed that not wearing condoms provided greater emotional pleasure which was presented as a significant benefit of unprotected sex. Only two participants believed that unprotected sex did not have emotional value. The rest felt that having unprotected sex provided a greater sense of emotional intimacy between partners, because it symbolised trust. Some participants said that unprotected sex was more special to them, as it was a symbol of commitment and investment in the relationship. For example: “*It’s a lot more than just [physical] pleasure. [Unprotected sex is] a lot more intimate because of that trust you have in someone. You want it to be like romantic and all about love, especially with a partner…. I feel like it shows that I trust you enough not to use condoms*” (Participant 6, male).

## 5. Conclusions

### 5.1. Condom Use and Trust

The research literature reviewed here identifies major factors affecting condom use as including self-efficacy, perceived loss of pleasure, shame, embarrassment, guilt, comfort communicating, “impurity” and lack of trust or emotional intimacy. In line with this, participants in this study reported that they would not discuss condom use with a partner they did not feel comfortable with, anticipated loss of pleasure was identified as the biggest disadvantage of condom use, and greater emotional intimacy as an advantage of unprotected sex. However, we also found that, with greater age or sexual experience, participants’ levels of confidence or self-efficacy were reported to increase, making them more likely to voice their desire for condom use to a partner.

In contrast to previous research in the West, this study demonstrates that using condoms with long-term partners is not thought to imply distrust or reduced emotional intimacy. Rather, all participants acknowledged that condoms are a sensible and safer choice. However, the more participants trusted their partners and the longer the relationship, the less likely they were to use them. Thus, unprotected sex with partners was understood as implying a deeper level of trust. Condom use however was not perceived as a sign of distrust but rather a non-offensive, logical measure of safety. Regardless of positive attitudes toward condom use, the likelihood of participants discussing their desire to use condoms depended on their comfort with their partner. Unlike previously found, norms set by parental and societal taboos were not identified as influencing condom use. Participants acknowledged that using condoms should not be stigmatised, and all participants identified it as a healthy behaviour. However, they did reveal the operation of stigma by admitting that they feared an awkward or harmful partner reaction when discussing condoms. It is important to note that this psychosocial stigma is different than the societal taboos and homophobia that studies found impacted condom use in the past studies. Here, it related to sexual double standards and ideas about trust.

Condom use depended on the level of trust participants had in their partner, regardless of length or type of relationship. Previous research found that participants would consistently use condoms in hook-up relationships. Although participants of this study said they were also more likely to wear condoms in hook-up or casual relationships, the decision to use condoms depended on the “trust” they had in their partners. Even in a hook-up situation, if they perceived their partners to be of “principled character” or they trusted them somewhat, then participants would not use condoms. We can conclude therefore that it is possible that either (UK) university students differ from the overall population regarding their ability to discuss and use condoms, or that these students were not representative of students in general, which in a study so small we would never try to claim anyway. What we do find interesting—and concerning—are the dynamics that we turn to now briefly.

### 5.2. Silencing: The Gender Dynamics of Heterosex

Like the findings of previous research, societal gender roles and gendered relationship dynamics are significant factors in shaping condom use by university students. Previous research suggests that traditional notions of femininity may be detrimental to women’s sexual health, as gender roles have been linked to lower sexual self-efficacy and assertiveness [38]. Traditional notions of femininity emphasize women’s passivity, compliance and agreeableness [39,40,41]. As a result of gender socialization, women experience greater forms of both internal and external silencing. Internal silencing involves internal barriers, such as uneasiness saying no, lack of confidence, fear of upsetting others, and the sense a woman might have that she should subordinate her desires to those of others, preventing her from taking control or voicing her desires. This means that even young women who value and aspire to equality do not always ask for their sexual needs to be met or for the safer sex they want to have in heterosexual encounters [42,43]. External silencing is when women who do voice their desires are silenced by others and by the consequences of speaking out. In feminist analyses, imposed silence is a tool of disempowerment and is used to control others [44].

The findings of this study illustrate unequal power relations between men and women still today. Traditional notions of femininity and relationship dynamics still greatly influence condom use, as many of the subthemes identified resulted in the silencing of women. Only the female participants reported experiencing silencing. Subthemes such as trust, comfortability with partner, and perceived hurtful partner reaction highlight the difficulties women face when it comes to voicing their desire to use condoms. When women were passive and did not use a condom, it was not because they did not want to, but because their expectations of response prevented them from voicing their desires. Although, in some cases, male participants also experienced internal barriers that caused them anxiety, they never reported silencing themselves for the sake of their partner. In many cases, when female participants were able to surpass their internal barriers and voice their sexual health concerns, they were met with external silencing in the form of guilting and lying. External silencing, the most overt method of trying to exert control over a partner and their choices, was only experienced by female participants. Both external and internal silencing effectively keep women from taking control of their health and illustrate the unequal power relations between men and women today and the dynamics that heterosexual women need to navigate.

### 5.3. New Forms of External Silencing: Stealthing as IPV

This study demonstrates that dishonesty about condom use is now a factor in unprotected sex amongst young people. In the literature reviewed for this study, stealthing was not mentioned or identified as a factor that impacted condom use behaviour. However, in this small study, two of the three male participants described stealthing as a prevailing practice amongst men they knew and identified it as a factor in unprotected sex. Only one female participant discussed stealthing, as she had experienced it herself. The other female participants did not refer to it.

Stealthing is another gender-related facilitator of unprotected sex, which highlights the unequal power relations between men and women today. Stealthing occurs more frequently to women than men, as demonstrated both in this and previous [45] research. Survivors of stealthing explain that it feels like a violation of trust and a denial of autonomy [25]. Stealthing takes away sovereign control of bodies, by silencing or overriding someone’s desire to protect their sexual health and often is also over-ruling a woman’s fertility decisions. Thus, stealthing is another form of IPV. In Lorde’s [44] formulation externally imposed silence seeks to produce powerlessness. In this era of third and fourth wave feminism, where women are unapologetically taking control of their sexual and reproductive health, stealthing is a tactic to silence women and keep them from finding their power. This is illustrated in Participant 4’s experience (subtheme: Lies and Misguided Trust). It was not enough that she had decided to use condoms to protect her sexual health and had voiced this desire to her partner. She was still silenced, as she was ignored by the partner, who wanted to be “defiant” of her. This illustrates that he wanted to exert power over her, by disrespecting her decisions and superseding them with his own. More generally, where male participants identified stealthing as a common tactic used by men against women, the findings of this study highlight an unequal power relationship between men and women, where men attempt to maintain control by silencing women or not respecting their decisions.

The findings of this study are limited by several factors. Participants may not have provided completely honest accounts, given the sensitivity of the topic. It is possible that they may have withheld information or adjusted their response to be more socially desirable. Participants described condom use behaviours retrospectively, so may not have recalled all the factors that influenced their decisions accurately. The small study group of nine students is too small to extrapolate to UK university students in general of course, as they are diverse in terms of sexual practice, heritage and country of origin. However, small studies can identify processes or dynamics even though they cannot quantify them, and these findings extend previous research by identifying new factors that influence condom use amongst university students. They also accentuate previous findings on women’s negotiation of their preferences for sexual safety. Understanding the meanings attributed to condom use by university students is necessary in order to inform educational interventions and policies. Stealthing and deceitful sexual practices need be included in research to illuminate such practices further, in order to raise awareness of them in professional training and therefore to improve sexual health and welfare support, and in sexual health education.

This study provides a glimpse of what affects the decision-making of university students regarding condom use. These nuances in decision-making might not be captured in quantitative studies, and so further, qualitative, studies of the factors that impact condom use amongst students are needed. Better understanding of stealthing can highlight the power dynamics in which sexual health and reproductive decisions are made by heterosexually active women, and spotlight this very intimate and private form of IPV. More research is needed around stealthing, its impact on sexual health and its reach by feminist-informed sexualities education or learning around sexual ethics.

## Figures and Tables

**Table 1:** List of themes and sub-themes identified from 9 interviews.

Themes	Subthemes
Experiences and Education	Formal Education Personal Experience Friend Experiences
Perceptions of Condom Purpose	Condoms Used as Contraception Condoms Used as STI Prophylactic
Communication	Trust and Comfortability Communicating with Partner Lies and Misguided Trust Approachability Relationship Type
Social and Psychological Pressures	Perceived Hurtful Partner Reaction Partner Guilting Stigma and Fear of “Ruining the Moment” Stereotypes
Decision-Making Effort	Comprehensive Decision Making Lack of Critical Thinking
Pleasure	Physical Emotional

STI—sexually transmitted infection.

## Data Availability

The data is not publicly available, as permission was not granted for archiving.
